# Progress in Ophthalmology

**Published:** 1904-01-09

**Authors:** 


### Progress in Ophthalmology.

(*Concluded from page* 246.)

**Subconjunctival Injection of Tuberculin in the Treatment of Interstitial Keratitis.**[3]—Darier quotes a most interesting case of interstitial keratitis which he believed to be of a tuberculous nature both on account of the absence of all signs of syphilis, and from the violent reaction caused by the tuberculin. The patient, a girl, was aged 13, her teeth were good, and she had a scrofulous appearance. The parents were healthy, and of eight children only one died of a tuberculous affection. In 1899 she had typhoid fever, and in 1900 there were repeated attacks of inflammation in the right eye. In a year the sight was completely destroyed, and the year following the left eye became affected. There was interstitial keratitis of a sclerosing form, and some injection. The right eye appeared to be lost, the iris invisible, but she had still perception of light. At first oily collyria with essence of clove—alternating in applications of dionine in powder and massage of the cornea with mercurial lanoline—were tried, but without effect. Jequiritol was next tried, at first with great reaction, but the eye became immune to the drug and did not react further; by this time patient could see motion of hand and various colours. During this period the left eye underwent various changes. She tolerated subconjunctival injections of cyanide of mercury, but soon a fresh relapse occurred. Hetol as a subconjunctival injection produced no reaction, but had a good influence in clearing up the morbid processes. He then, in the right eye, used a subconjunctival injection of tuberculin. The local reaction was most violent, but there was no rise of temperature. For eight days there was intense chemosis, and pannus developed. A considerable number of injections were given until February, 1903, when the cornea had become transparent enough to allow the contents of the anterior chamber to be seen with an abundant exudation on the back of Descemet's membrane. The fluid in the anterior chamber was so viscid it could not be aspirated. Posterior synechiæ were present, and an iridectomy was performed on March 15th. By June the vision had returned, the right being 1-15 and the left 1-18. This case illustrates that the opinion so many surgeons hold that interstitial keratitis is not necessarily due to syphilis is a good one.

**The Present Status of Subconjunctival Injections in Ophthalmic Therapeutics.**[4]—In a paper on this subject read before the New York Ophthalmological Society by Charles Steadman Bull, some valuable conclusions are drawn. At the beginning he went through the history of this method of treatment during the past seven years, and says an examination of all the papers published on this subject, by the various authors quoted, and a careful study of all the cases of all kinds reported leads irresistibly to at least one conclusion—that the efficiency of these solutions injected beneath the ocular conjunctiva cannot be ascribed to the increased local acceleration of the lymph currents, the so-called leucocytosis, nor to the antiseptic action of the remedies employed. Since the presence of such processes cannot be demonstrated in the tissues of the eye, following the injections, the chief change seems to be in the composition of the aqueous humour. This is said to become much richer in albuminoids, due to the irritating action of the injected substances upon the blood-vessels. This causes congestion of the vessels and an increased transudation of albuminoid substances from the vessels into the aqueous humour. The physiological chemists have taught us that the protective substances of the blood are always to be found in combination with the albuminoids of the blood. Hence coincidental with the increase of serum albumin in the aqueous humour would be the appearance of various protective substances in the same. He then proceeds to his own experience in the matter, and reports that he has employed various solutions, sodium chloride, sublimate, mercuric cyanide, and hetol, this last a sodium cinnamate. He has not been able to determine any important difference in their mode of action or effect, between salt solutions and solutions of mercuric-cyanide, so highly extolled by Darier. He has found this latter very painful to the patient, even when cocaine or acoin was added to the solution before injection. Hetol or sodium cinnamate was employed in a 1-per-cent. aqueous solution, of which 0·5 grm. was at first injected every second day, cocaine being first injected. This was used in various corneal affections, herpes, wounds, pannus, interstitial keratitis, as well as in choroiditis and scleritis. This solution was less painful than the mercurial preparations, and seemed to influence favourably the course of the disease in herpes and interstitial keratitis. It was used persistently in two cases of tuberculous disease of the iris and choroid, but apparently without the slightest effect. He sums up by saying: My own conclusions, based on observations of my own cases and a careful study of the literature of the subject, are, that all reports of the beneficial effects of subconjunctival injections should be carefully criticised, and compared with the results obtained by other methods of treatment, before accepting them as of any real value.

**The Need of a Supplementary Lantern Test for Proper Examination of Colour Vision.**—Charles H. Williams[5] thinks that a very extended use of the Holmgren test in the hands of many examiners is now showing that some cases are able to pass this test with skeins of coloured worsted correctly and without hesitation, who, when examined with the lights from a distant signal, may be unable to distinguish the red and green with any reasonable degree of certainty, and, in fact, often confuse these colours. This he attributes most frequently to cases of acquired defect in colour-vision caused by alcohol-tobacco amblyopia. As is well known, this trouble begins only with small scotomata for red and green, and Williams thinks the skeins of wool with which the test is made are large enough to cover the scotoma and a good portion of the surrounding retina, so that its colour is readily recognised. He advocates a test with lights of varying sizes, and also—and it seems to us most important—of varying intensity; this latter of course to correspond with the lights seen through fog, rain, or driving snow, or through steam issuing from the funnels of engines. Williams has devised an electric lamp which carries out these conditions. It struck us very forcibly that the electric lamp used in ophthalmoscopy and made by Curry and Paxton would, with a suitable cover and discs, be a very efficient method of working this test, and the Board of Trade of this country would be well advised to have such a contrivance in their testing stations.

[3] Brit. Med. Journ., Sept. 26, 1903. [4] Med. Rec., July 18, 1903. [5] Ibid., Aug. 8, 1903.

